# Prediction of ground reaction forces and moments during walking in children with cerebral palsy

**DOI:** 10.3389/fnhum.2023.1127613

**Published:** 2023-03-08

**Authors:** Julie Kloeckner, Rosa M. S. Visscher, William R. Taylor, Elke Viehweger, Enrico De Pieri

**Affiliations:** ^1^Laboratory for Movement Biomechanics, Department of Health Science and Technology, Institute for Biomechanics, Swiss Federal Institute of Technology (ETH) Zurich, Zurich, Switzerland; ^2^Department of Biomedical Engineering, École Polytechnique Fédérale de Lausanne (EPFL), Lausanne, Switzerland; ^3^Department of Biomedical Engineering, University of Basel, Basel, Switzerland; ^4^Laboratory for Movement Analysis, University Children’s Hospital Basel (UKBB), Basel, Switzerland

**Keywords:** kinetics, ground reaction forces (GRFs), musculoskeletal modeling, cerebral palsy, gait analysis

## Abstract

**Introduction:**

Gait analysis is increasingly used to support clinical decision-making regarding diagnosis and treatment planning for movement disorders. As a key part of gait analysis, inverse dynamics can be applied to estimate internal loading conditions during movement, which is essential for understanding pathological gait patterns. The inverse dynamics calculation uses external kinetic information, normally collected using force plates. However, collection of external ground reaction forces (GRFs) and moments (GRMs) can be challenging, especially in subjects with movement disorders. In recent years, a musculoskeletal modeling-based approach has been developed to predict external kinetics from kinematic data, but its performance has not yet been evaluated for altered locomotor patterns such as toe-walking. Therefore, the goal of this study was to investigate how well this prediction method performs for gait in children with cerebral palsy.

**Methods:**

The method was applied to 25 subjects with various forms of hemiplegic spastic locomotor patterns. Predicted GRFs and GRMs, in addition to associated joint kinetics derived using inverse dynamics, were statistically compared against those based on force plate measurements.

**Results:**

The results showed that the performance of the predictive method was similar for the affected and unaffected limbs, with Pearson correlation coefficients between predicted and measured GRFs of 0.71–0.96, similar to those previously reported for healthy adults, despite the motor pathology and the inclusion of toes-walkers within our cohort. However, errors were amplified when calculating the resulting joint moments to an extent that could influence clinical interpretation.

**Conclusion:**

To conclude, the musculoskeletal modeling-based approach for estimating external kinetics is promising for pathological gait, offering the possibility of estimating GRFs and GRMs without the need for force plate data. However, further development is needed before implementation within clinical settings becomes possible.

## 1. Introduction

Cerebral palsy (CP) is the most common motor disability in childhood, with a prevalence of 1 per 1,000 live births in Europe (Arnaud et al., 2018). In spastic CP, which is the most common form of CP, symptoms such as tremor, hypertonia, and limb weakness are often reported (Gage et al., 2009; Forni et al., 2018). To deal with these symptoms, individuals with CP present diverse compensatory strategies during walking, leading to pathological gait patterns (Rodda and Graham, 2001). To understand these compensatory strategies and identify their causes, instrumented clinical gait analysis (CGA) has become increasingly commonplace, and is also used for supporting clinical decision making regarding treatment planning and monitoring (Armand et al., 2016).

During CGA in children with CP, kinematic and kinetic information is collected by means of optical motion capture, 3D tracking systems, and ground reaction force (GRF) plates (Armand et al., 2016). However, to enable a complete understanding of the patient-specific motor impairment, quantification of the internal joint kinetics is required (Sloot and van der Krogt, 2018), which can be estimated using inverse dynamics analysis (Derrick et al., 2020). Here, the musculoskeletal system is generally modeled as a rigid body system starting from the acquired kinematic data (skin-mounted marker trajectories), from which segment and joint center locations and velocities, as well as linear and angular accelerations are derived. The inertial forces associated with the motion are then computed based on assumptions regarding the inertial characteristics of the body segments (Dumas and Wojtusch, 2018), while additional information on the external forces applied to the body is required before a complete dynamic characterization of the motion can be achieved. During CGA, this information can be obtained through force plates integrated into the floor, which commonly provide the magnitude, orientation, and point of application of the GRF vector. These data can also be equivalently reported in the form of three force and three moment vectorial components [ground reaction forces and moments (GRF&Ms)] in a force plate based coordinate system.

Collecting GRF&Ms remains challenging, even in advanced laboratory settings. Here, targeting clean force plate hits might change the natural movement pattern and corresponding measured kinetics (Challis, 2001). Force plates are also subject to error, e.g., hysteresis of the sensors, linearity errors, electrical inductance, and signal interference, which can all affect the estimated external kinetics (Psycharakis and Miller, 2006; Pamies-Vila et al., 2012). Finally, force plates are rarely available in external, unconstrained, environments, which limits measurements in real-life settings (Dorschky et al., 2019). As a result, a number of techniques to predict GRF&Ms solely from kinematic input data have recently emerged (Oh et al., 2013; Fluit et al., 2014; Skals et al., 2017; Dorschky et al., 2019; Lim et al., 2020). One of the more promising techniques is based on musculoskeletal modeling (Smith et al., 2021), in which the external GRF&Ms can be calculated based on the need for all segment forces to balance those of the body’s motion (Andersen, 2021; De Pieri et al., 2022c). Previous investigations using these techniques to predict GRF&Ms within the AnyBody Modeling System (AMS, AnyBody Technology, Aalborg, Denmark) have shown promising results in healthy subjects, with Pearson correlation coefficients of 0.80 and higher (Fluit et al., 2014; Skals et al., 2017). While such predictive models have been tested in patients with orthopedic (Peng et al., 2018; Zhang et al., 2020; Oh et al., 2021) and neurological (Eltoukhy et al., 2017) disorders, it remains unknown how they perform for locomotor pathologies that include toe-walking, such as children with CP who are the primary beneficiaries of CGA, where the outcome can influence clinical decision-making. Specifically, capturing GRF&Ms in children with CP can be extremely challenging as they often use walking aids or take very small steps, hence, making the collection of clean force plate hits, where only one whole foot completely contacts the force plate, difficult. Therefore, the goal of our study was to investigate if GRF&Ms can be predicted from pathological gait kinematics to a similar level achieved for asymptomatic gait patterns. In addition, we aimed to estimate the influence of predicted GRF&Ms on the resulting ankle, knee, and hip joint moments, toward understanding the suitability of applying these methods to support clinical decision making.

## 2. Materials and methods

In this study, we firstly tested the reproducibility of previous results from healthy individuals (Fluit et al., 2014; Skals et al., 2017) in a pathological cohort affected by CP. Next, model performance was assessed for both the unaffected and affected limbs in hemiplegic gait patterns. Finally, the effect of predicted vs. measured GRF&Ms on the calculation of joint moments was quantified to provide an understanding of the applicability of this method for determining internal joint contact forces and for supporting clinical decision-making.

### 2.1. Participants

For this observational study, CGA data were retrospectively extracted from the patient database of a local hospital. Participants were included in the study if they were between 6 and 18 years of age at time of measurement, diagnosed with unilateral spastic CP, and underwent routine gait analysis between August 2019 and April 2022. Patients were excluded if they had a gross motor function classification system (GMFCS) level worse than I (Rosenbaum et al., 2002), underwent any surgical intervention within the year prior to CGA, received a botulinum toxin type A injection within 6 months prior to measurement, or had missing clinical files. The participants were split into five subgroups according to their spastic hemiplegic gait patterns (Rodda classification; Rodda and Graham, 2001). Patients’ data were screened, starting with the most recent cases, until five participants were included in each subgroup. The final dataset therefore consisted of 25 participants (Table 1).

**TABLE 1:** Participant demographics.

	Type 1	Type 2A	Type 2B	Type 3	Type 4
Age (years)	11.8 ± 2.3	10.5 ± 3.8	11.2 ± 1.9	11.3 ± 4.6	12.3 ± 3.3
Gender (m/f)	2/3	4/1	3/2	4/1	3/2
Height (cm)	155.5 ± 16.4	141.5 ± 25.9	138.7 ± 12.3	145.5 ± 22.1	153.3 ± 23.4
Weight (kg)	50.3 ± 21.5	41.9 ± 22.4	35.8 ± 10.3	40.4 ± 13.2	40.8 ± 19.6

All participants were diagnosed with unilateral spastic cerebral palsy with a gross motor function classification scale (GMFCS) level I. Gait patterns were classified according to Rodda and Graham (2001), type 1: drop foot, type 2A: true equinus, type 2B: true equinus/recurvatum knee, type 3: true equinus/jump knee, type 4: equinus/jump knee with pelvic rotation and hip flexed, adducted, and internally rotated. Values represent mean ± SD, except for gender which is given in count.

### 2.2. Experimental procedure

**FIGURE 1:**
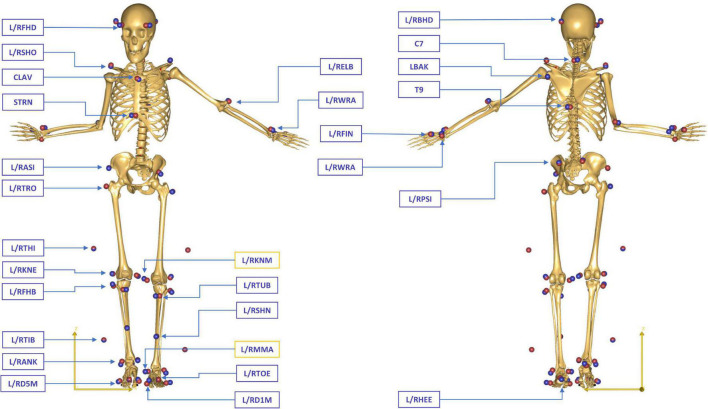
Full-body marker set of the conventional gait model (CGM 2.2) during a static standing reference trial in a patient with CP. The blue spheres represent the location of the physical markers measured in the motion laboratory, while the red spheres indicate the position of the virtual markers attached to the skeletal template in AMS. The generic model was scaled for each subject to match the overall anthropometrics and marker data collected during the static standing reference trial. Medial epicondyle (L/RKNM) and malleolar (L/RMMA) markers were only included in the static trials, while all other markers were used for the tracking of both static and dynamic trials.

Instrumented gait analysis was performed using an optoelectronic motion capture system with 12 cameras (MXT20, Vicon, Oxford Metrics Ltd., UK) at a sampling rate of 150 Hz. The full-body marker set of the conventional gait model (CGM 2.2, 9.5 mm diameter markers, Vicon, Oxford Metrics Ltd., UK) (Leboeuf et al., 2019) was used to collect the kinematic data (Figure 1). Four force plates (sampling frequency 1,500 Hz, Kistler Instrumente AG, Winterthur, Switzerland) embedded in the laboratory floor were used to collect kinetic information.

At the start of the measurement, a clinical examination was performed by a trained physiotherapist during which each patient’s anthropometrics such as height, weight, leg length, and femoral anteversion were measured. Afterward, a static trial in a standing reference pose with abducted shoulders was acquired. Each participant was then asked to walk barefoot over the 12 m instrumented walkway until six valid stride cycles for each leg were captured, i.e., when only one foot contacted each force platform, without stepping over its edges. Trials with excessive soft tissue artifact, poor consistency, or signs of inaccurate marker placement were excluded.

Markers data were labeled, gap-filled, and filtered (Woltring filter, mean squared error set to 10 mm^2^) using Vicon Nexus (version 2.8.2, Vicon, Oxford Metrics Ltd., UK) (Woltring, 1986). Initial contact and toe-off events were determined from the GRF measurements using force thresholds (>20N for initial contact and <20N for toe-off).

### 2.3. Musculoskeletal modeling

Measured marker trajectories and GRF&Ms were used as input for an inverse dynamics analysis in the AnyBody Modeling System (AMS, AnyBody Technology A/S, Aalborg, Denmark) (Damsgaard et al., 2006), based on the AnyBody Managed Model Repository (AMMR, v.2.3.3). GRF&Ms were filtered using a second-order low-pass Butterworth filter with a cut-off frequency of 12 Hz. Personalized models for each subject were created from a detailed generic model of the lower limb (De Pieri et al., 2018) based on a reference cadaveric dataset (Carbone et al., 2015) and scaled to match the overall anthropometrics and marker data collected during the static standing reference trial (Lund et al., 2015; Figure 1). The geometry of the femur was linearly morphed to include a transversal rotation between the proximal and distal sections, matching the subject’s femoral anteversion obtained from the clinical assessment (De Pieri et al., 2021; Alexander et al., 2022). The hip joints were modeled as 3 degrees-of-freedom (DoFs) ball-and-socket joints, while the knee, talocrural, and subtalar joints were modeled as 1-DoF hinges. Additionally, the position of the patella was defined as a function of the knee flexion angle (Carbone et al., 2015). The 166 muscle elements in each leg were modeled as constant strength actuators.

A kinematic analysis based on the marker trajectories was conducted to compute joint kinematics (Andersen et al., 2009; Lund et al., 2015). Mean marker tracking error was additionally computed as the mean distance between each pair of measured and virtual markers’ positions during the whole gait cycle and averaged across all full-body markers for each patient. Subsequently, two different inverse dynamics analyzes were performed. The first analysis took the GRF&Ms measured from the force plates as input, while in the second, these quantities were predicted solely from the 3D full-body motion, based on a dynamic contact model and optimization techniques (Fluit et al., 2014; Skals et al., 2017). In both analyzes, the muscle recruitment problem was solved through static optimization by minimizing the sum of the squared muscle activations in order to calculate the required muscle activations and forces, as well as resulting joint moments (Andersen, 2021).

### 2.4. GRF&M prediction

**FIGURE 2:**
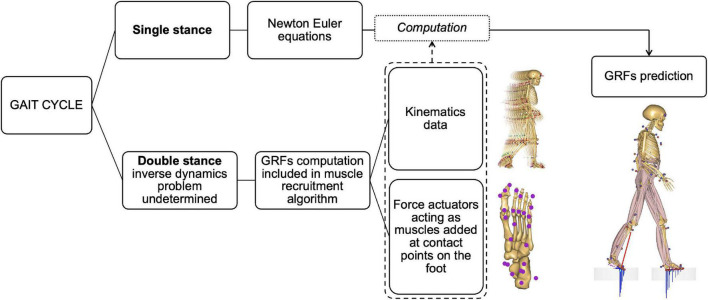
Diagram of the method for predicting ground reaction forces and moments (GRF&Ms) in the different phases of the gait cycle. In single stance, the equations of motion allow the direct calculation of GRF&Ms from kinematics. During double stance, the problem is underdetermined and does not allow the direct calculation of load distribution between the two limbs. The estimation of GRF&Ms is therefore included in the muscle recruitment algorithm via contact nodes under the foot acting as muscles to balance the body model during motion. The combination of these nodes with kinematic data allows the calculation of GRF&Ms.

It is possible to predict GRF&Ms from measured full-body kinematic data and estimated mass and inertial properties of the subject within AMS by adding conditional contacts that act as force actuators connecting the feet to the ground while satisfying the Newton–Euler equations of motion (Fluit et al., 2014; Skals et al., 2017). These contact points generate the normal and frictional forces necessary to kinetically balance the model at each instant in time. During single support phases, GRF&Ms can be computed directly by solving the Newton–Euler equations, provided that the full-body kinetics and kinematics are available. During double support phase, in which the system defines a closed kinetic chain with the ground, GRF&Ms can be computed by predicting the forces generated by the foot-ground actuators as part of the optimization of the muscle recruitment problem, thus not requiring any training or empirical data (Fluit et al., 2014; Figure 2).

**FIGURE 3:**
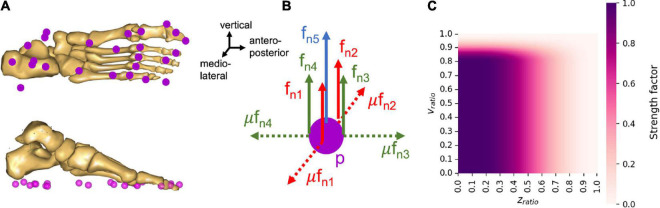
Properties of the nodes under the foot to predict GRF&Ms. **(A)** Organization of the contact nodes under the foot, **(B)** actuator organization for each contact node, p, where *f_n_* is the shear force normal to the contact plane, and μ is the coefficient of friction. **(C)** Proportion of the maximum strength available for a node at different heights and velocity ratios.

In each scaled musculoskeletal model, 25 nodes are created under each foot (Figure 3). Each of these nodes consists of five unilateral force actuators, allowing reaction forces to push the foot off the ground, as well as friction components characterized by a static Coulomb friction model. Four of these force actuators are organized into pairs such that normal forces *f_n_* and their corresponding friction forces μ*f*_*n*_ can be generated in a positive or negative (antero-posterior or medio-lateral) direction corresponding to the foot contact planes. The friction coefficient μ was set to 0.5, which corresponds to the coefficient measured during walking (Vidal et al., 2021). The last force actuator, aligned with the vertical axis, can generate only a force normal to the surface (Figure 3B). The sum of these 5 actuator forces corresponding to each node are then calculated as part of the muscle recruitment problem (Rasmussen et al., 2001; Forster et al., 2004; Damsgaard et al., 2006; Fluit et al., 2014), in other words, contact nodes are recruited in the same manner as muscles.

The strength profile of the force actuators at each node is defined as a function of the distance and velocity between the node and the ground. This strength profile reflects the fact that the force actuators can only be recruited, and therefore generate forces, when in stationary contact with the ground, i.e., when static equilibrium can be assumed. The contact of a node with the ground is therefore considered when the node is located under a certain height *z*_*lim*_, and moves below a certain speed *v*_*lim*_. If these conditions are not met, the available strength for the corresponding node is zero. When a contact is detected, the available strength then changes from zero to maximum, hence, creating a discontinuity in the force profile of the node. In order to avoid such discontinuities, a sinusoidal smoothing function was added to the force actuator strength function, depending on the ratios of node position divided by maximum height and of node velocity divided by maximum speed of contact detection:


(1) cp,i=Nmaxif zratio≤0.8 and vratio≤0.15Nsmooth if 0.8≤zratio≤1 and 0.15≤vratio≤10otherwise

$$z_{r\,a\,t\,i\,o}=\frac{p_{z}}{z_{l\,i\,m}}$$

$$v_{r\,a\,t\,i\,o}=\frac{p_{v\,e\,l}}{v_{l\,i\,m}}$$

where *c*_*p,i*_ is the strength profile of the *i^th^* node at position *p*, *N*_*max*_ is the maximal strength a node can generate and was set to 40% of body weight, *N*_*smooth*_ is the smoothed profile of the strength function, *z*_*ratio*_ is the vertical position of the node divided by the maximal height for contact detection, *v*_*ratio*_ is the velocity of the node divided by the maximal speed for contact detection, with *z*_*lim*_ set to 0.03 m and *v*_*lim*_ set to 0.8 m/s. The smoothed profile of the strength function (*N*_*smooth*_) was defined as follows:


(2) $$N_{s\,m\,o\,o\,t\,h}=N_{m\,a\,x}\,z_{s\,m\,o\,o\,t\,h}\,v_{s\,m\,o\,o\,t\,h}$$

$$z_{s\,m\,o\,o\,t\,h}=\frac{1}{2}\,\left(1+\cos\left(\frac{\left(z_{r\,a\,t\,i\,o}-0.8\right)\,\pi }{1-0.8}\right)\right)$$

$$v_{s\,m\,o\,o\,t\,h}=\frac{1}{2}\,\left(1+\cos\left(\frac{\left(v_{r\,a\,t\,i\,o}-0.15\right)\,\pi }{1-0.15}\right)\right)$$

The strength profile thus describes the progressive level of node strength as a function of the node’s height and speed ratios (Figure 3C). The non-linear formulation of the strength profile, the sinusoidal smoothing function, as well as the contact thresholds for distance and velocity were adapted from the work of Fluit et al. (2014) and Skals et al. (2017).

To account for dynamic inconsistencies between the GRF&M measurement data, the marker data, and the predicted internal forces within the musculoskeletal system, conventional musculoskeletal modeling workflows usually apply balancing residual forces at the pelvis (known as the “Hand of God” in AMS). In order to predict the GRF&Ms from full-body kinematic data and conditional foot-ground contacts, these residual forces need to be deactivated. Instead, weak muscle-like actuators are added to the pelvis to ensure dynamic consistency of the modeled motion and prevent the simulation from failing due to out-of-balance forces in the system. These muscle-like actuators are also recruited as part of the muscle recruitment optimization problem; however, their low strength ensures that their contribution remains minimal, therefore only contributing to the numerical stability of the analysis (Fluit et al., 2014). Finally, mean magnitude of the residual force and moment vectors at the pelvis during the gait cycle were computed, using GRF&M measurement data as input.

### 2.5. Data analysis

Gait trials were processed and analyzed through the toolkit AnyPyTools (Lund et al., 2019). For each investigated stride cycle, all GRF&M vectors were transformed into a consistent global reference frame, aligned with the direction of gait, to ensure comparability between the measured and predicted results. Internal joint moments for the hip, knee, and ankle were calculated in their respective proximal segment coordinate systems according to ISB recommendations (Derrick et al., 2020), with variables of clinical interest reported.

One representative trial per subject was then selected by calculating the average curve for each plane of all trials that captured a complete gait cycle on both limbs. The average root-mean-square deviation (RMSD) of each trial was compared to the averaged curve, where the trial with the smallest RMSD was chosen as the most representative. GRF&Ms and joint moment trajectories were then time-normalized to the gait cycle (GC) from foot-strike (0%) to foot-strike (100%) of the leg of interest, and additionally normalized to the body mass of each subject.

### 2.6. Statistical analysis

To evaluate quality of the prediction algorithm, predicted GRF&Ms were compared to those measured using force plates for both the affected and unaffected limbs. Differences were quantified through RMSD, cross-correlation coefficient, and Pearson correlation coefficient (PCC) to allow comparison to previous studies in healthy individuals (Fluit et al., 2014; Skals et al., 2017), where PCC values were categorized as weak (≤0.35), moderate (0.36–0.67), strong (0.68–0.9), or excellent (>0.9) (Taylor, 1990). In addition, Statistical Parametric Mapping (SPM, v.0.4.8, spm1d-package^1^) paired *t*-tests were used to test if there were significant differences between predicted and measured GRFs across the gait cycle, with significance set at α < 0.05 (Pataky, 2010). To compare performance between spastic hemiplegic gait patterns, RMSD and PCC values for the affected legs were calculated per sub-group.

To investigate the influence of GRF&M predictions on estimates of joint moments, joint moments were calculated using the predicted GRF&Ms and compared to those determined based on force platform data (SPM paired *t*-tests).

## 3. Results

### 3.1. Ground reaction forces and moments

**FIGURE 4:**
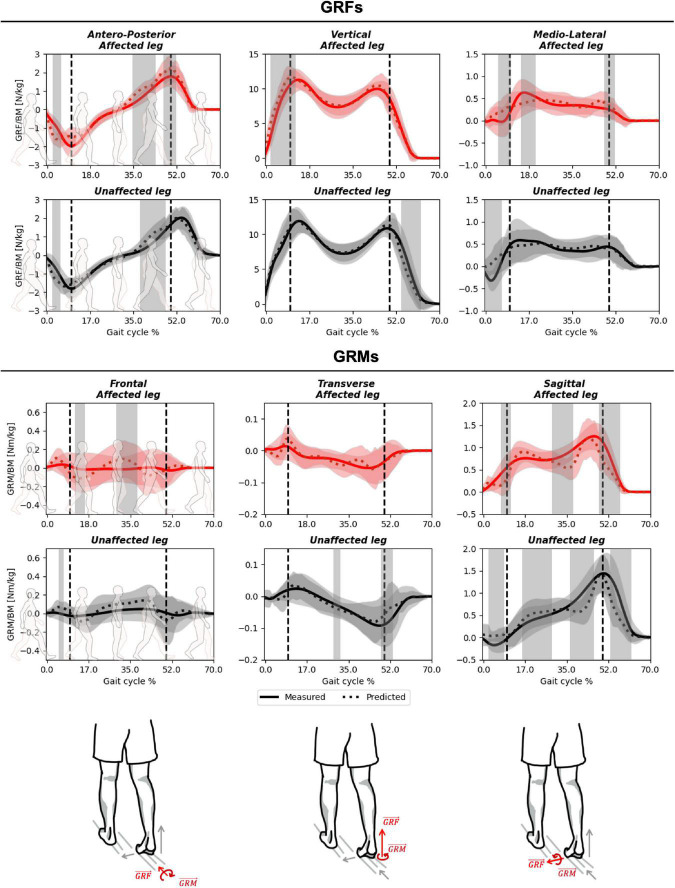
Measured against predicted GRF&Ms over the course of a gait cycle for affected (red) and unaffected (black) limbs in children with spastic unilateral cerebral palsy. The outcomes for GRFs **(top)**, GRMs **(middle)**, and planes from left to right antero-posterior/frontal, vertical/sagittal, and medio-lateral/transverse **(bottom)** are shown. Gray shading highlights significant differences (*p* < 0.05) detected using statistical parametric mapping paired *t*-test. The vertical dashed lines indicate average transitions between single and double stance phases.

The predicted and measured GRFs presented comparable trends throughout the gait cycle in the antero-posterior and vertical planes (Figure 4 and Supplementary Figure 1), with excellent PCC values of 0.94–0.96 (Supplementary Figure 1). In the mediolateral axis, where the GRF component was smallest, the predicted signal exhibited a lower PCC (average 0.71–0.80) and the greatest relative RMSD (average 18–20 vs. 9–12% for the antero-posterior and vertical planes, Table 2 and Supplementary Figure 1). Compared to these GRFs, higher deviations were determined for the GRMs, with higher RMSDs (20–66%, Table 3), especially in the antero-posterior direction (64–66 vs. 10–12%), and lower PCC values (0.32–0.76, Supplementary Figure 1).

**TABLE 2:** Root-mean-square deviation of the measured and predicted GRF&Ms in the three gait axes and planes for affected and unaffected limbs of children with spastic unilateral CP.

		Unaffected			Affected		
		Antero-posterior	Vertical	Medio-lateral	Antero-posterior	Vertical	Medio-lateral
GRFs	RMSD (N/kg)	0.40 ± 0.09	1.04 ± 0.27	0.20 ± 0.06	0.46 ± 0.10	1.13 ± 0.25	0.18 ± 0.05
	RMSD (% of max magnitude)	10 ± 3%	9 ± 2%	18 ± 4%	12 ± 3%	10 ± 2%	20 ± 6%
		**Frontal**	**Transverse**	**Sagittal**	**Frontal**	**Transverse**	**Sagittal**
GRMs	RMSD (Nm/kg)	0.12 ± 0.06	0.03 ± 0.01	0.30 ± 0.09	0.12 ± 0.02	0.03 ± 0.01	0.28 ± 0.06
	RMSD (% of max magnitude)	66 ± 39%	24 ± 13%	20 ± 4%	64 ± 46%	33 ± 14%	21 ± 3%

Values are given in the standard unit (N/kg or Nm/kg) and as the percentage of the maximum amplitude. GRFs are predicted with a low error (8–20%), but GRM errors are considerably higher (up to 63%). Values represent mean ± SD. RMSD, root-mean-square deviation.

**TABLE 3:** Rootmean-square deviations (RMSDs) of joint moments in the sagittal and frontal planes derived from predicted vs. measured GRF&Ms for unaffected and affected limbs of children with spastic unilateral CP.

		Unaffected			Affected		
		Hip	Knee	Ankle	Hip	Knee	Ankle
Sagittal	RMSD (Nm/kg)	0.19 ± 0.05	0.17 ± 0.04	0.26 ± 0.12	0.21 ± 0.04	0.19 ± 0.05	0.24 ± 0.09
	RMSD (% of max magnitude)	15% ± 6%	17% ± 9%	17% ± 9%	17% ± 5%	25% ± 11%	24% ± 10%
Frontal	RMSD (Nm/kg)	0.14 ± 0.06	0.10 ± 0.05		0.13 ± 0.05	0.11 ± 0.03	
	RMSD (% of max magnitude)	13% ± 7%	19% ± 11%		17% ± 8%	26% ± 12%	

Values are given in the standard unit (N/kg or Nm/kg) and as the percentage of the maximum amplitude. The propagation of predicted GRF&Ms errors to the ankle, knee, and hip might still influence clinical decisions. RMSD, Root mean square deviation. Values represent mean ± standard deviation.

SPM analysis revealed that, in the antero-posterior direction, significant differences between measured and predicted GRFs were observed at initial contact and during terminal stance, for both unaffected and affected limbs (Figure 4). In both cases, the magnitude of the predicted GRF was greater than that of the measured GRF. In the vertical direction, significant differences were observed at the pre-swing phase, for the unaffected limb, and at initial contact for the affected limb. While forces were generally underestimated for the unaffected limb, they were mostly overestimated for the affected side. In the medio-lateral direction, a significant overestimation was identified at initial contact for both limbs. No phase shifts were present between measured and predicted GRF&Ms for any of the planes or limbs (Supplementary Figure 1). For the GRMs, significant differences were observed in the frontal plane during midstance to terminal stance in both limbs, while in the transverse plane, this significant difference was only present in the unaffected limb. For the sagittal plane, significant differences were present at terminal stance in both limbs. Additionally, differences between measured and predicted GRMs were present at initial contact, midstance, and pre-swing for the unaffected limb in the vertical direction.

When specifically examining differences within spastic hemiplegic gait patterns, both measured and predicted GRF&Ms were very similar (Supplementary Figure 2 and Supplementary Tables 1, 2), with average relative RMSDs for GRFs variating between 10 and 15% in the antero-posterior direction, 9–10% in the vertical direction, and 18–22% in the medio-lateral direction. For GRMs, differences between the spastic hemiplegic groups ranged from 55 to 71% in the frontal plane, 17 to 29% in the transverse plane, and from 18 to 22% in the sagittal plane.

### 3.2. Joint moments

**FIGURE 5:**
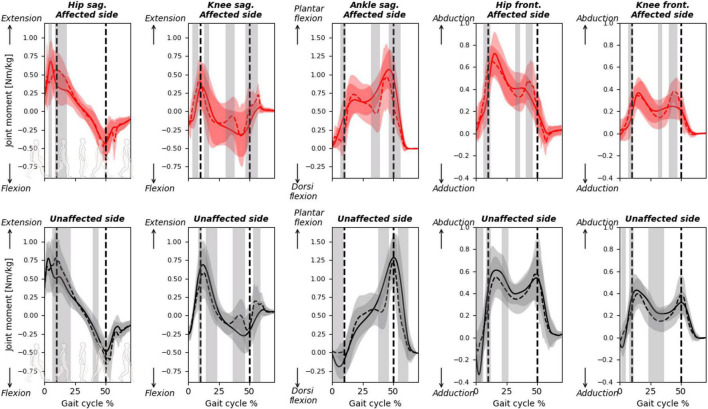
Joint moments calculated with predicted or measured GRF&Ms over the course of a gait cycle for affected (red) and unaffected (black) limbs in children with spastic unilateral CP. Hip and knee moments in the sagittal (flexion-extension, **top**) and frontal (abduction-adduction, **bottom**) planes, while ankle moments in the sagittal plane (dorsiflexion-plantarflexion, **top**) are shown. Gray shading highlights significant differences (*p* < 0.05) detected using statistical parametric mapping paired *t*-test. The vertical dashed lines indicate average transitions between single and double stance phases.

The RMSDs for the ankle, knee, and hip joint moments calculated between the predicted and measured GRF&Ms were all similar, with averages between 13 and 26% and average PCCs between 0.70 and 0.92 (Table 3 and Supplementary Figure 3). Deviations between measured and predicted joint moments were highest at terminal stance and pre-swing for the knee and ankle joints in the sagittal plane (Figure 5 and Supplementary Figure 3). On the unaffected side, significant differences are also detected during initial contact for the ankle joint in the sagittal, and for the hip joint, in the frontal plane.

## 4. Discussion

Ground reaction forces and moments has been successfully predicted in research settings for assessing human kinetics during various activities of daily living (Fluit et al., 2014; Jung et al., 2017; Skals et al., 2017). This prediction of GRF&Ms is able to support our understanding of muscle requirements during different complex motions, or to estimate joint loading conditions in real life scenarios. However, the applicability of such approaches for supporting clinical decision-making in subjects with pathological gait patterns, and specifically toe-walkers, has not yet been investigated. In the current study, we evaluated the performance of an available GRF&Ms prediction algorithm on spastic hemiplegic gait patterns in children with CP. For both the affected and unaffected legs, differences between predicted and measured GRF&Ms were comparable to those observed in healthy individuals (Fluit et al., 2014). The performance of the prediction algorithm was also similar across different spastic hemiplegic gait patterns. The levels of error found in the determination of joint moments derived from the predicted GRF&Ms, however, suggest that caution should still be taken when using such approaches in clinical decision-making.

In general, the vertical component of the GRF and sagittal plane GRM were best predicted by the algorithm, with strong to excellent PCC, which is in-line with previous findings (Fluit et al., 2014; Skals et al., 2017). While PCC for antero-posterior and medio-lateral components of the GRFs were also strong to excellent (0.71–0.95, Table 2 and Supplementary Figure 1), the corresponding GRM correlations in the frontal and transverse planes were only weak to moderate (0.31–0.43, Table 2 and Supplementary Figure 1). The largest deviations between predicted and measured GRMs were found in the transverse plane, which also agrees with previous reports (Fluit et al., 2014). The relatively higher error observed for the GRF&M components with lower magnitudes is plausibly due to the increased signal-to-noise ratio resulting from soft-tissue artifact (Leardini et al., 2005). The absolute errors found in this study were, however, slightly higher than values reported in literature (GRFs our study vertical: 1.09 ± 0.22 vs. 0.74 ± 0.13 N/kg, antero-posterior: 0.45 ± 0.09 vs. 0.38 ± 0.07 N/kg, medio-lateral: 0.18 ± 0.05 vs. 0.17 ± 0.04 N/kg, GRMs sagittal: 0.27 ± 0.06 vs. 0.18 ± 0.05 Nm/kg), which is not entirely surprising as previous investigations mainly comprised of healthy individuals (Fluit et al., 2014).

Sharp peaks were observed within the predicted GRF&M components during the transition from double to single stance and vice-versa, hence, resulting in a more discontinuous appearance than actual measured forces and moments. This discontinuity of the predicted signals was more prominent than in previous reports, probably because previous investigations have presented the average of multiple activity trials instead of reporting only single trials, thereby artificially “smoothing” the predicted signals. Interestingly, these discontinuities were observed equally for both the unaffected and affected limbs, as well as across different spastic hemiplegic gait patterns, likely due to the approach used to distribute GRF&Ms across two limbs in such closed-chain scenarios. During these phases of double support, the predicted GRF&Ms are computed as part of the muscle recruitment problem, hence, finding the most optimal solution. The optimality of this solution, however, might not hold true for individuals with pathological gait patterns, who could have impairments in controlling their motion optimally, or could choose to move with different motor control strategies. For example, CP subjects commonly present sub-optimal muscle activation patterns, due to e.g., contractures or spasticity (Kim et al., 2018a), or might move with the goal of avoiding pain rather than maintaining efficient motion (Veerkamp et al., 2019). Different control strategies could also lead to an imbalance in the relative loading patterns between limbs. Here, no large differences were observed in performance of the GRF&M prediction algorithm between the sides of the hemiplegic spastic gait patterns (Figure 4). However, the vertical component of the GRF on the affected limb was overestimated during initial contact, but underestimated contralaterally during pre-swing. No such difference between sides was reported when applying the prediction algorithm on healthy walking patterns (Fluit et al., 2014). It is possible that this side-to-side difference results from the non-optimality of the pathological locomotor patterns, as children with unilateral CP rely more on their unaffected limb than on their affected one, especially during double support phases, potentially due to a lack of strength and confidence in their affected limb (Kim et al., 2015).

To estimate GRF&Ms during movement, a full-body marker protocol, including upper extremities, trunk, and head, with a sufficient number of markers to characterize all segment motions, is required. From this kinematic data, a musculoskeletal model reproducing the captured motion is created. Typical modeling assumptions and simplifications, such as a rigid foot segment, a 1-DOF hinge joint at the knee, and a rigid trunk segment, might limit the capacity of the model to represent the real kinematics, thus leading to errors in the prediction of the internal kinetics of the musculoskeletal system. In this study, we observed a mean marker tracking error of 1.4 cm across all subjects (range: 1.1–1.9 cm). Similarly, segments’ dimensions and inertial properties are linearly scaled within the AMS musculoskeletal models, using an adult cadaver as a reference (Carbone et al., 2015). The assumption that such an adult healthy subject is representative of the anatomy and segmental inertial properties of pathological children could introduce considerable errors in the computation of internal forces during the reproduced motion (Camomilla et al., 2017; Hainisch et al., 2021). During a typical AMS inverse dynamics analysis based on force plate data, all kinetic mismatches between the reproduced motion of the scaled human model and the measured GRF&Ms are compensated by artificial actuators located at the pelvis, which balance the kinetics of the modeled motion. When using force plate data as input, we found the mean magnitude of the residual force and moment vectors at the pelvis across all patients to be 0.81 N/kg (range: 0.44–1.61) and 0.25 Nm/kg (range: 0.17–0.30), respectively. Within the workflow to predict GRF&Ms, these actuators are necessarily deactivated, and therefore any kinetic mismatch (error in internal forces) is propagated to the predicted GRF&Ms. This could partially explain some of the observed differences between measured and predicted signals. Future studies should devise segment-specific and gait-phase-specific marker-tracking and force residual metrics to assess potential improvements in experimental marker protocols and modeling assumptions, such as joint DoFs and segmental inertial properties. In particular, the simplified foot model, which cannot account for foot bending (e.g., mid-foot break) that is a key characteristic of pathological gait patterns (Stebbins et al., 2010), could also potentially influence the predicted position of the contact nodes with respect to the ground. A more detailed foot model would likely go a long way toward mitigating these limitations (Kim et al., 2018b). Similarly, more realistic knee and thoracic models could improve the accuracy of the predictions (Fluit et al., 2014; Ignasiak et al., 2016; Dejtiar et al., 2020).

The current results did not indicate a difference in performance of the GRF&M prediction algorithm across different spastic hemiplegic gait patterns, which presented rather consistent RMSD values. This might be due to the fact that only high functioning children with CP (GMFCS level I) were included in this investigation. Although toe-walking was predominant in our population, their gross motor function impairments were only low, and not as severe as can be expected in children with GMFCS level II. It should be noted, however, that the rather small sample size (*n* = 5 unilateral CP subjects per category) limits the possibility to perform sub-group statistical analysis. It would therefore be interesting to further assess the impact of specific locomotor impairment patterns on prediction performance, including varying degrees of severity, with larger sample sizes.

While the GRFs were relatively well predicted, errors between measured and predicted GRMs were relatively high, indicating that the algorithm had difficulties identifying the location at which the force vector acted. These errors in GRMs propagated toward the moments at the ankle, knee, and hip joints, to an extent that could influence clinical decisions. Pathological gait patterns in CP are identified through a number of kinematic and kinetic features (Brunner and Rutz, 2013; Sloot and van der Krogt, 2018). For instance, toe-walking gait patterns are often characterized by a double-peak profile of the ankle plantar flexion moment, and an erroneous prediction of these profiles (Figure 5, unaffected ankle sagittal moment) could misinform clinical decisions. Furthermore, the peak ankle plantar flexion moment during push-off provides information on the severity of toe-walking and serves as an indicator of the effectiveness of certain treatments (Sloot and van der Krogt, 2018). Predicting GRF&Ms led to an underestimation of the peak moment, which could translate into erroneous clinical conclusions. Deviations in sagittal knee moments in patients compared to controls can be used in CGA to infer quadriceps weaknesses, patellar pain, or quadriceps overload (Armand et al., 2016; De Pieri et al., 2022c). Furthermore, the knee adduction moment is often used as a surrogate measure for the load distribution between the medial and lateral compartments of the knee (Holder et al., 2020; De Pieri et al., 2022b), while reduced hip abduction moment in patients can be associated with a functional deficit of the hip abductors in the presence of altered femeral morphologies (Thielen et al., 2019; Alexander et al., 2022; De Pieri et al., 2022a). However, significant differences between the two methods in the computed sagittal and frontal moments for both knee and hip between the two methods suggest that this these metrics should be interpreted carefully while predicting GRF&Ms. Overall, a more accurate prediction of the joint moment profiles, as well as their peak values, is warranted to translate these predictive algorithms into trustworthy diagnostic tools and to expand the applicability of musculoskeletal modeling in clinical settings (Smith et al., 2021), particularly for subjects with CP.

## 5. Conclusion

In conclusion, our study demonstrates that musculoskeletal modeling-based approaches to predict GRF&Ms are able to estimate external kinetics for affected and unaffected limbs in different spastic hemiplegic gait patterns, with error levels similar to those previously reported for gait in healthy individuals. By estimating GRF&Ms solely based on kinematics, without the need for measured force plate data, this approach therefore presents potential to expand biomechanical investigations beyond confined motion laboratories into real-life scenarios (Larsen et al., 2020; Skals et al., 2021). Moreover, the approach can be combined with novel motion-tracking technologies such as inertial measurement units (IMUs), depth-sensor cameras, and markerless techniques (Karatsidis et al., 2017; Ripic et al., 2022), to offer less-constrained application possibilities. Although promising, the error levels for the derived joint moments might still influence clinical decisions, and further improvements are required before the approach can be reliably translated into clinical settings.

## Data availability statement

The datasets analyzed for this study can be found in the ETH Research Collection (doi: 10.3929/ethz-b-000597686). Further inquiries can be directed to the corresponding author.

## Ethics statement

The studies involving human participants were reviewed and approved by the Cantonal Ethics Committee Zurich (KEK 2018-01640). Written informed consent to participate in this study was provided by the participants’ legal guardian/next of kin.

## Author contributions

RV and EDP: conceptualization and data curation. JK: investigation and visualization. JK, RV, and EDP: methodology and writing—original draft. JK and EDP: software. JK and RV: formal analysis. RV, WT, EV, and EDP: project administration. WT, EV, and EDP: supervision. JK, RV, WT, EV, and EDP: writing—review and editing. All authors contributed to the article and approved the submitted version.

## Footnotes

1
http://www.spm1d.org/
