# Supplementary material for: Prediction of ground reaction forces and moments during walking in children with cerebral palsy
Source: Front Hum Neurosci. 2023 Mar 8;17:1127613. doi: 10.3389/fnhum.2023.1127613 (PMC10031015; doi:10.3389/fnhum.2023.1127613)
Supplement: Supplementary file 1 [file Table_1.DOCX]

|  | | | **Unaffected** | | | **Affected** | | |
| --- | --- | --- | --- | --- | --- | --- | --- | --- |
|  |  |  | **Antero Posterior** | **Vertical** | **Medio Lateral** | **Antero Posterior** | **Vertical** | **Medio Lateral** |
| **GRFs** | **Rodda 1** | **RMSD [N/kg]** | 0,41 ± 0,06 | 0,98 ± 0,19 | 0,19 ± 0,03 | 0,46 ± 0,07 | 0,99 ± 0,13 | 0,18 ± 0,02 |
|  |  | **RMSD [%]** | 12% ± 4% | 9% ± 3% | 19% ± 6% | 15% ± 3% | 9% ± 1% | 22% ± 7% |
|  | **Rodda 2A** | **RMSD [N/kg]** | 0,38 ± 0,10 | 0,97 ± 0,20 | 0,16 ± 0,02 | 0,44 ± 0,07 | 1,06 ± 0,11 | 0,20 ± 0,07 |
|  |  | **RMSD [%]** | 9% ± 2% | 8% ± 2% | 15% ± 2% | 11% ± 2% | 9% ± 1% | 18% ± 5% |
|  | **Rodda 2B** | **RMSD [N/kg]** | 0,43 ± 0,06 | 1,01 ± 0,29 | 0,18 ± 0,07 | 0,49 ± 0,19 | 1,19 ± 0,27 | 0,19 ± 0,07 |
|  |  | **RMSD [%]** | 10% ± 1% | 8% ± 2% | 19% ± 4% | 11% ± 4% | 10% ± 2% | 20% ± 4% |
|  | **Rodda 3** | **RMSD [N/kg]** | 0,34 ± 0,07 | 1,13 ± 0,40 | 0,17 ± 0,04 | 0,42 ± 0,08 | 1,11 ± 0,31 | 0,15 ± 0,02 |
|  |  | **RMSD [%]** | 9% ± 3% | 9% ± 3% | 15% ± 4% | 10% ± 2% | 9% ± 2% | 17% ± 5% |
|  | **Rodda 4** | **RMSD [N/kg]** | 0,40 ± 0,12 | 1,07 ± 0,31 | 0,24 ± 0,08 | 0,44 ± 0,09 | 1,12 ± 0,29 | 0,17 ± 0,05 |
|  |  | **RMSD [%]** | 9% ± 1% | 7% ± 1% | 18% ± 3% | 12% ± 5% | 10% ± 2% | 21% ± 5% |
|  | | | **Frontal** | **Transverse** | **Sagittal** | **Frontal** | **Transverse** | **Sagittal** |
| **GRMs** | **Rodda 1** | **RMSD [Nm/kg]** | 0,18 ± 0,09 | 0,03 ± 0,01 | 0,34 ± 0,03 | 0,12 ± 0,01 | 0,03 ± 0,00 | 0,29 ± 0,04 |
|  |  | **RMSD [%]** | 71% ± 4% | 20% ± 9% | 22% ± 4% | 85% ± 6% | 34% ± 18% | 21% ± 2% |
|  | **Rodda 2A** | **RMSD [Nm/kg]** | 0,10 ± 0,02 | 0,03 ± 0,01 | 0,27 ± 0,08 | 0,10 ± 0,03 | 0,04 ± 0,01 | 0,26 ± 0,04 |
|  |  | **RMSD [%]** | 58% ± 29% | 20% ± 6% | 18% ± 3% | 41% ± 16% | 28% ± 9% | 21% ± 2% |
|  | **Rodda 2B** | **RMSD [Nm/kg]** | 0,11 ± 0,03 | 0,03 ± 0,00 | 0,30 ± 0,08 | 0,11 ± 0,02 | 0,03 ± 0,00 | 0,28 ± 0,06 |
|  |  | **RMSD [%]** | 58% ± 30% | 22% ± 6% | 20% ± 4% | 71% ± 36% | 33% ± 9% | 21% ± 3% |
|  | **Rodda 3** | **RMSD [Nm/kg]** | 0,10 ± 0,03 | 0,03 ± 0,01 | 0,32 ± 0,09 | 0,10 ± 0,02 | 0,03 ± 0,01 | 0,30 ± 0,09 |
|  |  | **RMSD [%]** | 55% ± 48% | 17% ± 4% | 20% ± 4% | 69% ± 66% | 40% ± 18% | 21% ± 3% |
|  | **Rodda 4** | **RMSD [Nm/kg]** | 0,10 ± 0,02 | 0,03 ± 0,01 | 0,25 ± 0,05 | 0,13 ± 0,02 | 0,03 ± 0,01 | 0,23 ± 0,06 |
|  |  | **RMSD [%]** | 67% ± 34% | 29% ± 18% | 18% ± 3% | 48% ± 23% | 28% ± 10% | 21% ± 4% |

|  | | **Unaffected** | | | **Affected** | | |
| --- | --- | --- | --- | --- | --- | --- | --- |
|  |  | **Antero Posterior** | **Vertical** | **Medio Lateral** | **Antero Posterior** | **Vertical** | **Medio Lateral** |
| **GRFs** | **Rodda 1 PCC** | 0,95 ± 0,01 | 0,97 ± 0,01 | 0,73 ± 0,12 | 0,93 ± 0,01 | 0,96 ± 0,01 | 0,56 ± 0,29 |
|  | **Rodda 2A PCC** | 0,95 ± 0,02 | 0,96 ± 0,01 | 0,88 ± 0,03 | 0,95 ± 0,02 | 0,95 ± 0,02 | 0,68 ± 0,28 |
|  | **Rodda 2B PCC** | 0,95 ± 0,01 | 0,96 ± 0,02 | 0,73 ± 0,12 | 0,93 ± 0,03 | 0,95 ± 0,02 | 0,72 ± 0,12 |
|  | **Rodda 3 PCC** | 0,95 ± 0,03 | 0,96 ± 0,02 | 0,89 ± 0,05 | 0,95 ± 0,02 | 0,96 ± 0,02 | 0,80 ± 0,10 |
|  | **Rodda 4 PCC** | 0,96 ± 0,01 | 0,97 ± 0,01 | 0,76 ± 0,09 | 0,94 ± 0,02 | 0,95 ± 0,03 | 0,78 ± 0,05 |
|  | | **Frontal** | **Transverse** | **Sagittal** | **Frontal** | **Transverse** | **Sagittal** |
| **GRMs** | **Rodda 1 PCC** | 0,32 ± 0,40 | 0,48 ± 0,30 | 0,82 ± 0,16 | 0,06 ± 0,39 | 0,29 ± 0,47 | 0,77 ± 0,14 |
|  | **Rodda 2A PCC** | 0,48 ± 0,29 | 0,22 ± 0,60 | 0,18 ± 0,85 | 0,72 ± 0,32 | 0,08 ± 0,60 | 0,45 ± 0,41 |
|  | **Rodda 2B PCC** | 0,62 ± 0,30 | 0,63 ± 0,14 | 0,82 ± 0,14 | 0,77 ± 0,12 | 0,28 ± 0,28 | 0,79 ± 0,19 |
|  | **Rodda 3 PCC** | 0,46 ± 0,35 | 0,68 ± 0,34 | 0,83 ± 0,13 | 0,11 ± 0,65 | 0,27 ± 0,45 | 0,76 ± 0,17 |
|  | **Rodda 4 PCC** | 0,20 ± 0,74 | 0,33 ± 0,53 | 0,60 ± 0,78 | 0,58 ± 0,23 | 0,60 ± 0,36 | 0,91 ± 0,06 |
