# Supplementary figures and images for: Prediction of ground reaction forces and moments during walking in children with cerebral palsy
Source: Front Hum Neurosci. 2023 Mar 8;17:1127613. doi: 10.3389/fnhum.2023.1127613 (PMC10031015; doi:10.3389/fnhum.2023.1127613)

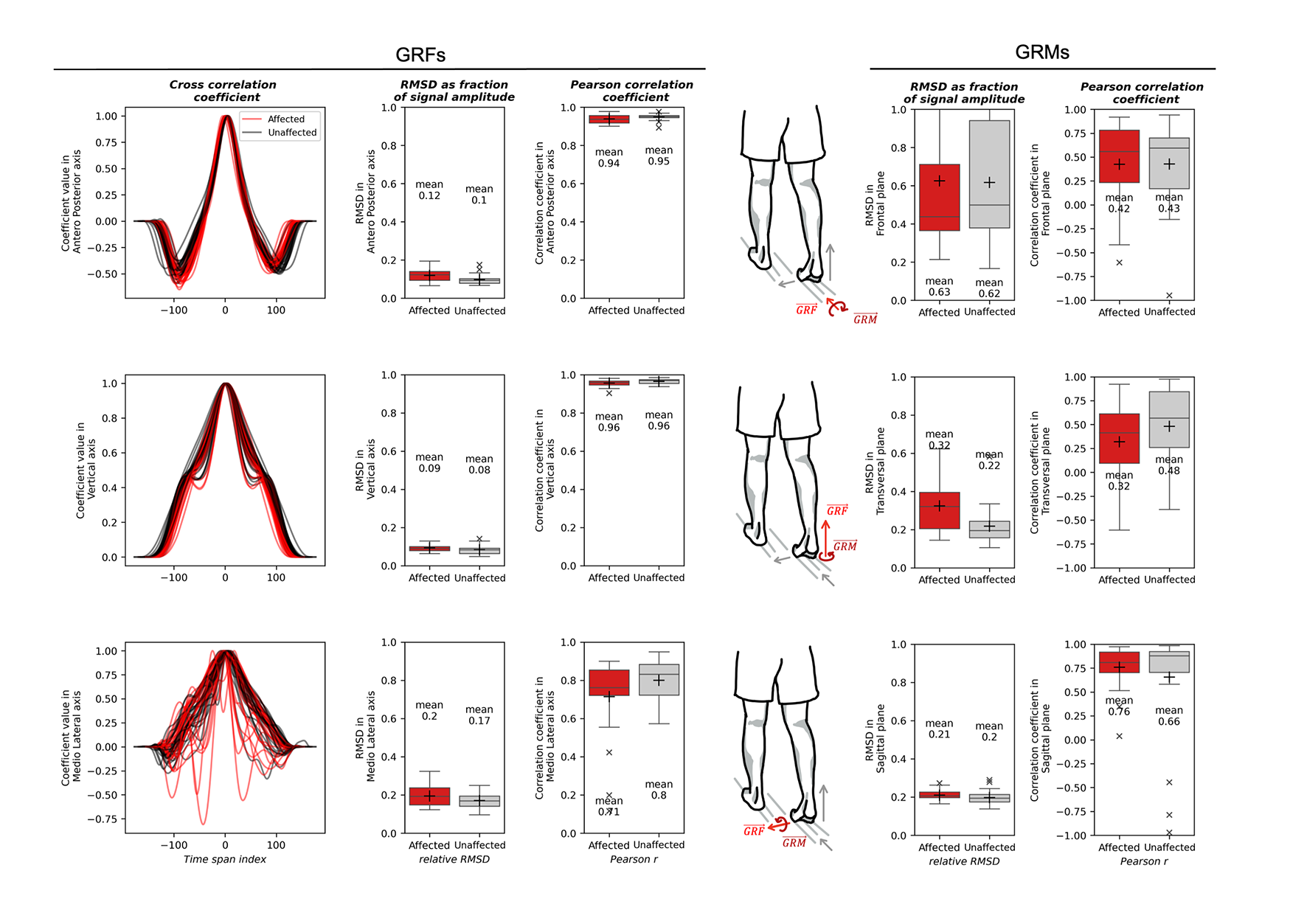

Supplement: Supplementary file 2 [file Image_1.TIFF]

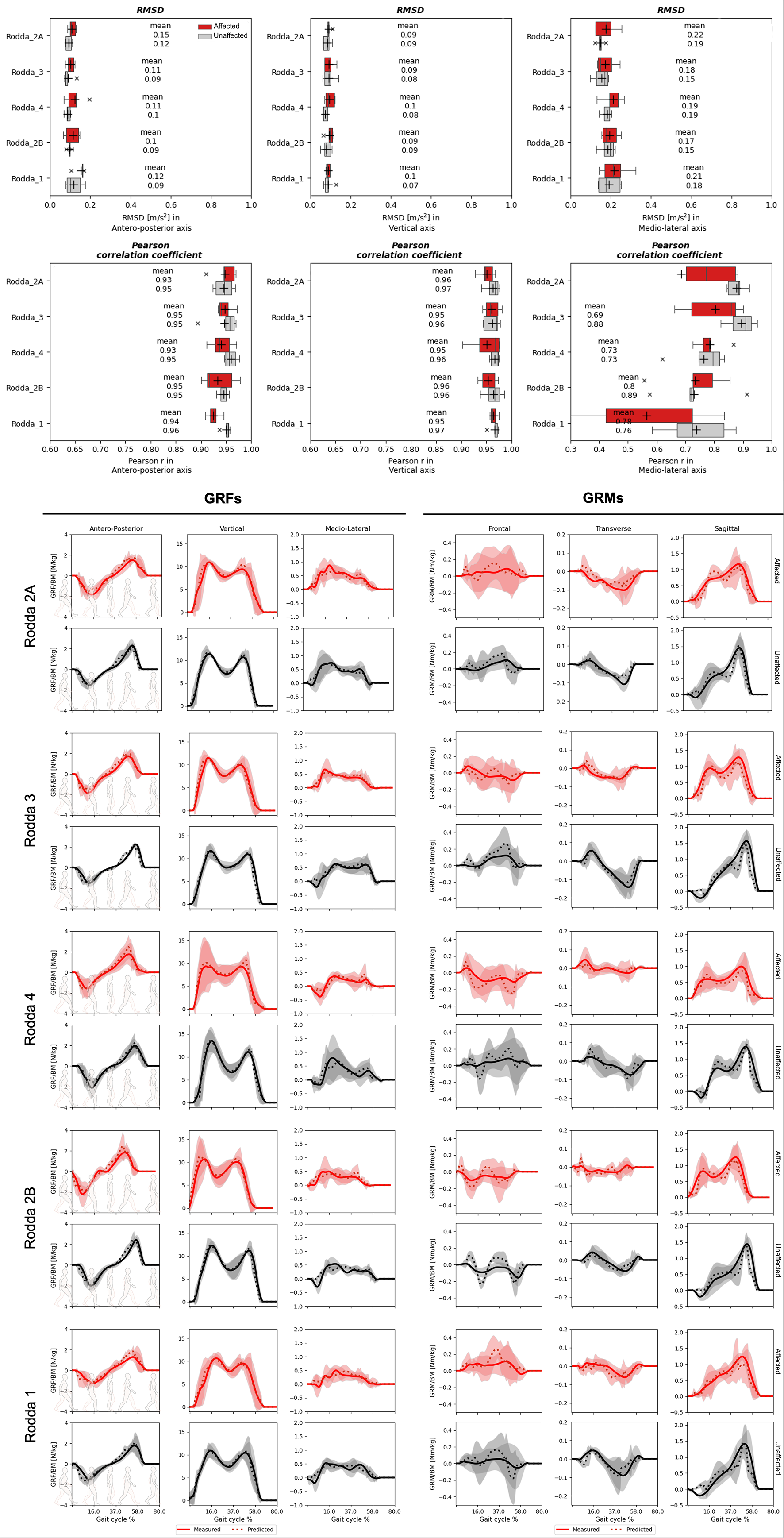

Supplement: Supplementary file 3 [file Image_2.TIFF]

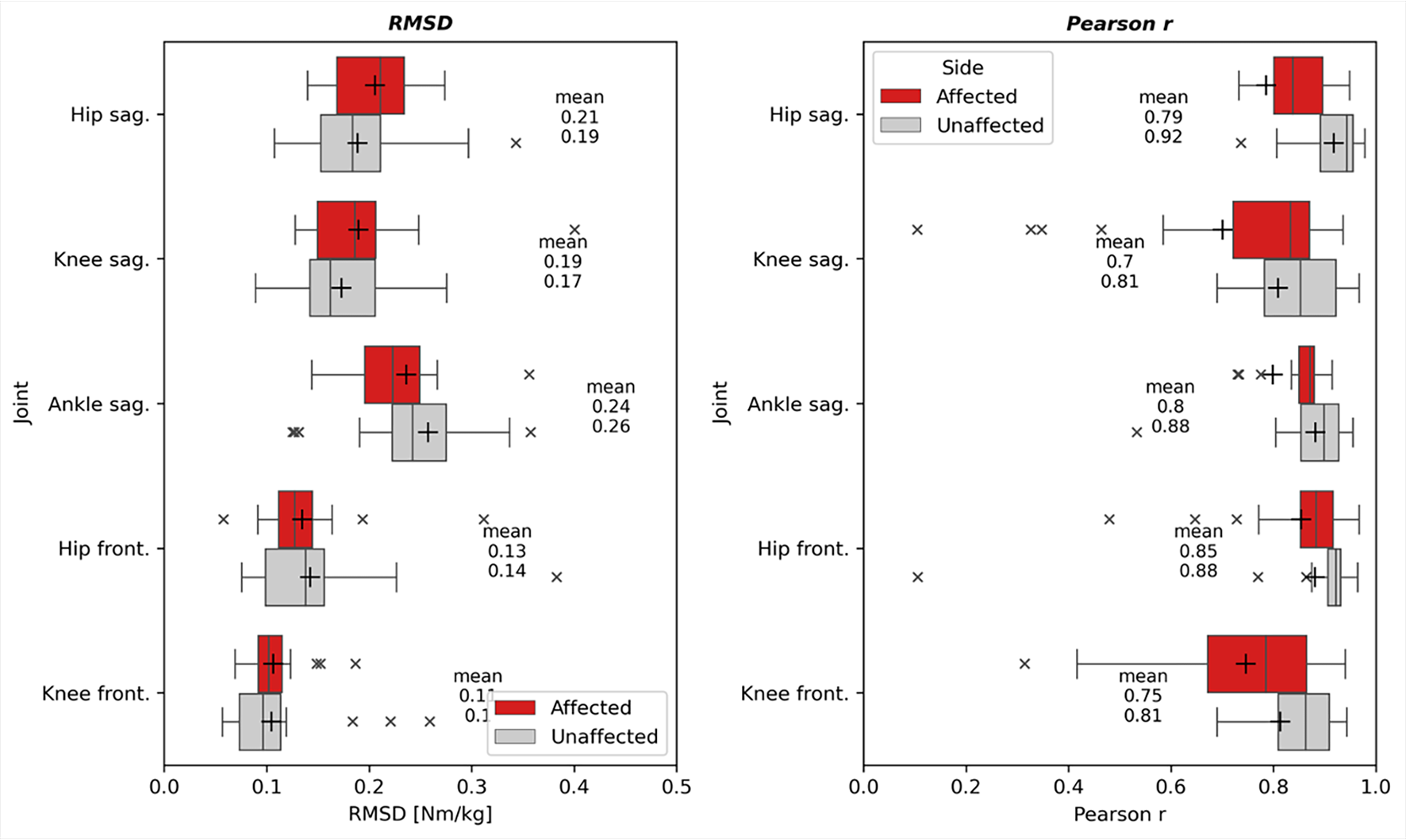

Supplement: Supplementary file 4 [file Image_3.tiff]
